# Motor unit recovery following Smn restoration in mouse models of spinal muscular atrophy

**DOI:** 10.1093/hmg/ddac097

**Published:** 2022-05-12

**Authors:** Laura H Comley, Rachel A Kline, Alison K Thomson, Victoria Woschitz, Eric Villalón Landeros, Erkan Y Osman, Christian L Lorson, Lyndsay M Murray

**Affiliations:** Centre for Discovery Brain Sciences, University of Edinburgh, Edinburgh, EH8 9XD, UK; Euan MacDonald Centre for Motor Neuron Disease Research, University of Edinburgh, Edinburgh, EH16 4SB, UK; Centre for Discovery Brain Sciences, University of Edinburgh, Edinburgh, EH8 9XD, UK; Euan MacDonald Centre for Motor Neuron Disease Research, University of Edinburgh, Edinburgh, EH16 4SB, UK; Centre for Discovery Brain Sciences, University of Edinburgh, Edinburgh, EH8 9XD, UK; Euan MacDonald Centre for Motor Neuron Disease Research, University of Edinburgh, Edinburgh, EH16 4SB, UK; Centre for Discovery Brain Sciences, University of Edinburgh, Edinburgh, EH8 9XD, UK; Euan MacDonald Centre for Motor Neuron Disease Research, University of Edinburgh, Edinburgh, EH16 4SB, UK; Bond Life Sciences Center, University of Missouri, Columbia, MO 65211, USA; Department of Biological Chemistry, Johns Hopkins University School of Medicine, Baltimore, MD 21205-2185, USA; Bond Life Sciences Center, University of Missouri, Columbia, MO 65211, USA; Department of Veterinary Pathobiology, College of Veterinary Medicine, University of Missouri, Columbia, MO 65211, USA; Bond Life Sciences Center, University of Missouri, Columbia, MO 65211, USA; Department of Veterinary Pathobiology, College of Veterinary Medicine, University of Missouri, Columbia, MO 65211, USA; Centre for Discovery Brain Sciences, University of Edinburgh, Edinburgh, EH8 9XD, UK; Euan MacDonald Centre for Motor Neuron Disease Research, University of Edinburgh, Edinburgh, EH16 4SB, UK

## Abstract

Spinal muscular atrophy (SMA) is a childhood motor neuron disease caused by anomalies in the SMN1 gene. Although therapeutics have been approved for the treatment of SMA, there is a therapeutic time window, after which efficacy is reduced. Hallmarks of motor unit pathology in SMA include loss of motor-neurons and neuromuscular junction (NMJs). Following an increase in Smn levels, it is unclear how much damage can be repaired and the degree to which normal connections are re-established. Here, we perform a detailed analysis of motor unit pathology before and after restoration of Smn levels. Using a Smn-inducible mouse model of SMA, we show that genetic restoration of Smn results in a dramatic reduction in NMJ pathology, with restoration of innervation patterns, preservation of axon and endplate number and normalized expression of P53-associated transcripts. Notably, presynaptic swelling and elevated Pmaip levels remained. We analysed the effect of either early or delayed treated of an antisense oligonucleotide (ASO) targeting SMN2 on a range of differentially vulnerable muscles. Following ASO administration, the majority of endplates appeared fully occupied. However, there was an underlying loss of axons and endplates, which was more prevalent following a delay in treatment. There was an increase in average motor unit size following both early and delayed treatment. Together this work demonstrates the remarkably regenerative capacity of the motor neuron following Smn restoration, but highlights that recovery is incomplete. This work suggests that there is an opportunity to enhance neuromuscular junction recovery following administration of Smn-enhancing therapeutics.

## Graphical abstract

**Figure:**
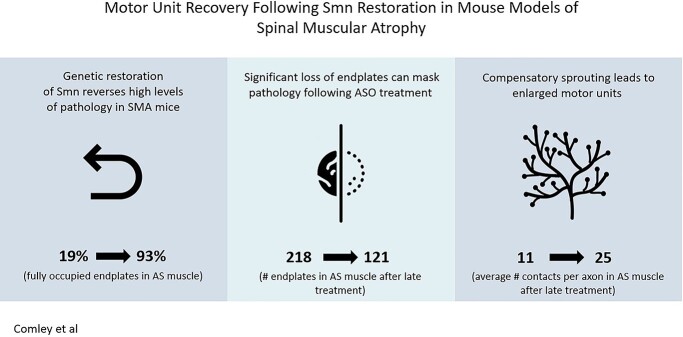

## Introduction

Spinal muscular atrophy (SMA) is a childhood motor neuron disease caused by mutations and deletions within the SMN1 gene (1). Due to a second partially functional copy of the gene, termed SMN2, which can exist in a variety of copy numbers, the severity of the disease varies. In its most severe form, SMA has an onset of under 6 months, with a life expectancy of $\sim$2 years. A number of therapeutics have recently been approved for the treatment of SMA that increase SMN protein levels, including an antisense oligonucleotide (ASO), a small molecule drug and viral gene therapy (2–5). Thus far, treatments have demonstrated remarkable efficacy, significantly slowing decline or greatly enhancing motor ability and lifespan in treated patients. However, we do not yet know the long-term prognosis following these treatments. It is likely that long-term outcomes will be closely linked to the amount of damage at the point of treatment and the degree to which motor neuron pathology can be reversed.

Preclinical studies have shown that there is a dramatic reduction in therapeutic efficacy when treatment is delayed (6–9). This therapeutic time window has been investigated in mouse models, and a delay in treatment of 1 or 2 days can result in a dramatic decrease in life expectancy (10–12). It is important to understand why a temporal window exists and develops strategies that extend this.

Although other tissues have been implicated in SMA pathology, motor neurons remain the primary targets (13) with pathology at the neuromuscular junction (NMJ) preceding motor neuron loss (14). Importantly, not all muscles appear to be equally affected, with high levels of NMJ loss observed in some muscles, whereas other muscles remain intact until late stages of disease (15–18). Although the patterns of selective vulnerability are highly stereotyped, and there are important parallels between patients and mouse models, the reasons for this underlying selective vulnerability remain unclear.

Early treatment with a Smn-inducing compound appears to reduce NMJ defects, with efficacy varying by the route of administration and level of Smn achieved (10–12,19–24). Improvements were seen in various aspects of NMJ pathology including increased quantal content, decreased neurofilament (NF) accumulation and increased endplate maturation. However, it is unclear whether early treatment prevents the NMJ defects from manifesting or corrects pathology that has already commenced.

It is well established that the peripheral nervous system is capable of significant regeneration. This is observed following an acute peripheral nerve injury, or as ongoing remodelling and compensation occurring during progressive peripheral neuropathies (25). Indeed, injured axons are able to regenerate and reinnervate denervated muscle. Intramuscular axons are also able to grow new branches from internodes (nodal sprouts) or from the synaptic terminal (terminal sprouts) to reinnervate endplates which have become denervated. These processes of compensation are major modifiers of disease time course. Indeed, extremely large motor units have been observed in patients, particularly with milder forms of SMA, where the few remaining motor neurons have sprouted and expanded their territory to compensate for the loss of their neighbours (26,27). However, it is unclear how motor unit expansion affects the overall health of the motor neuron, and it has been suggested that motor unit enlargement correlates with an increase in oxidative stress, which can affect the longevity of the cell (25).

It is currently unclear how pathology of the motor neuron is affected by restoration of Smn levels. For example, how much damage can be repaired effectively? Does restoration of Smn levels allow normal innervation patterns to be restored and is there a critical threshold of damage which occurs, which cannot be compensated for, that may explain the therapeutic time window in SMA?

Here, we have undertaken a detailed study of motor unit recovery following restoration of Smn levels. Using an Smn inducible mouse model of SMA, we show that at P4 [a time point where treatment with tamoxifen causes > 50% of mice to live over 200 days (11)], there are already high levels of NMJ pathology, including denervation, presynaptic swelling and an increase in transcripts associated with the P53 signalling pathway. Analysis of the pathology at P10 following Smn restoration at P4 reveals a remarkable recovery of the neuromuscular system within 6 days, suggesting that much of the pathology observed at P4 is rapidly reversible. Importantly, this recovery was achieved by reinnervation of denervated endplates and resumption of normal motor unit size, correlating with the predicted benefit to phenotype and lifespan following P4 rescue in this mouse model. To further investigate the basis for the effective therapeutic time window using a more clinically relevant approach, we treated ‘delta7’ mice (Smn^−/−^; SMN2^+/+^; SMNΔ7^+/+^) with a SMN-inducing ASO by intracerebral injection P1 (associated with a significant extension in lifespan) or P6 (associated with a limited extension in lifespan). Both early and late treatment with ASO led to very low levels of denervation. However, since there was also a reduction in total endplate number, the reduction in the number of denervated endplates can be partly attributed to the loss of these endplates rather than reinnervation. In moderately and severely affected muscles, there was also a loss of intramuscular axons and an increase in average motor unit size, which was more widespread following P6 treatment.

Collectively, this work highlights that rescue of the neuromuscular system is possible following restoration of SMN levels. However, it is incomplete and dependent on the method of treatment and the time point at which treatment is initiated. The long-term effects of incomplete rescue need to be further investigated to better understand how to support patients undergoing treatment for SMA.

## Results

### There are significant levels of NMJ pathology at P4 before Smn restoration in *Smn*^*Res/Res*^ mice

Smn inducible model mice (*Smn^Res/Res^; SMN2; SMN^Δ7^; Cre^ERT^*) have been described previously (11). They carry the *Smn^Res^* allele on the ‘delta7’ background. The *Smn^Res^* allele essentially functions as a null allele in the non-induced state, resulting in a severe SMA phenotype like the typical delta7 model. Administration of tamoxifen induces Smn expression, demonstrating that early induction significantly extends survival with over 50% of mice living over 200 days when Smn is induced at P4. It is unclear whether treatment at P4 prevents pathology manifesting, or whether regeneration/repair is required. To determine how much damage has been done to the neuromuscular system at this time point, we assessed phenotype and neuromuscular pathology at P4.

**Figure 1:**
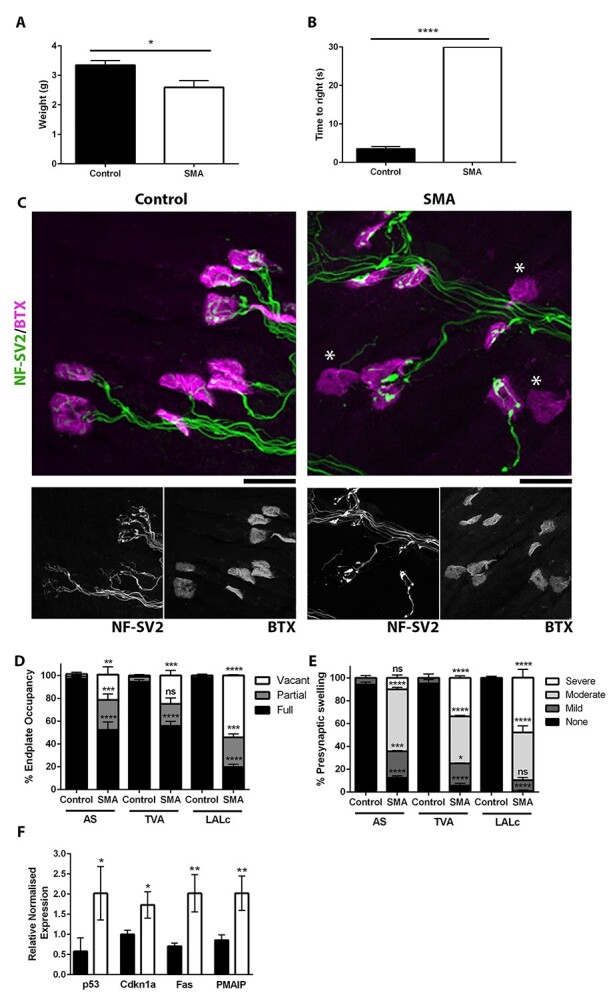
There is significant NMJ pathology at P4 in Smn^Res/Res^ mice before Smn restoration. (**A**, **B**) Bar chart (mean$\pm$SEM) showing significant decrease in body weight (A) and significant increase in time taken to self-right (B) in SMA (Smn^Res/Res^;SMN2;SMNΔ7) mice compared with control (Smn^+/Res^;SMN2;SMNΔ7 and Smn^+/+^;SMN2;SMNΔ7) at P4. (**C**) Images showing neuromuscular junctions (NMJs) in the TVA muscle labelled with antibodies against NF and SV2(green) and BTX (magenta) in control and SMA mice at P4. Note presence of denervated endplates (BTX lacking presynaptic terminal) in SMA which does not occur in control. Scale Bar = 16 μm (**D**) Bar chart (mean$\pm$SEM) showing percentage of fully occupied, partially occupied and vacant endplates in AS, TVA and Levator Auris Longus Caudal (LALc) muscles in control and SMA mice at P4. Note increase in vacant and partially occupied endplates in SMA and corresponding decrease in fully occupied endplates. (**E**) Bar chart (mean$\pm$SEM) showing percentage of NMJs with no, mild, moderate or severe swelling in AS, TVA and LALc muscles in control and SMA mice at P4. Note increase in presynaptic swelling in SMA and corresponding decrease NMJs with no swelling. (**F**) Bar chart (mean$\pm$SEM) showing increase in relative transcript levels of P53, Cdkn1a, Fas and Pmaip in the spinal cord of P4 SMA mice compared with control. ^*^P < 0.05; ^*^^*^P < 0.01, ^*^^*^^*^P < 0.001, ^*^^*^^*^^*^P < 0.0001 by Student’s *t* test (A, B) or Mann–Whitney U test (D, E, F). *N* = 3/4 mice, 6 muscles per genotype.

In agreement with previous work (11), in untreated mice at P4, we observed a significant reduction in body weight and motor deficits, as evidenced by an inability to self-right (Fig. 1A and B). We next quantified NMJ pathology in three muscles which have previously been shown to be vulnerable in other mouse models of SMA (17,28): auricularis superior (AS), transversus abdominis (TVA) and levator auris longus caudal band (LALc). This revealed high levels of denervation in all three muscles, with a significant increase in the percentage of partially occupied and vacant endplates, with only 19.7 (LALc)–55.9% (TVA) endplates remaining fully occupied (Fig. 1C and D). In those terminals which remained fully occupied, we saw a significant increase in presynaptic swelling, as evidenced by a significant increase in the percentage of NMJs with mild, moderate and severe swelling in all three muscles (Fig. 1E).

**Figure 2:**
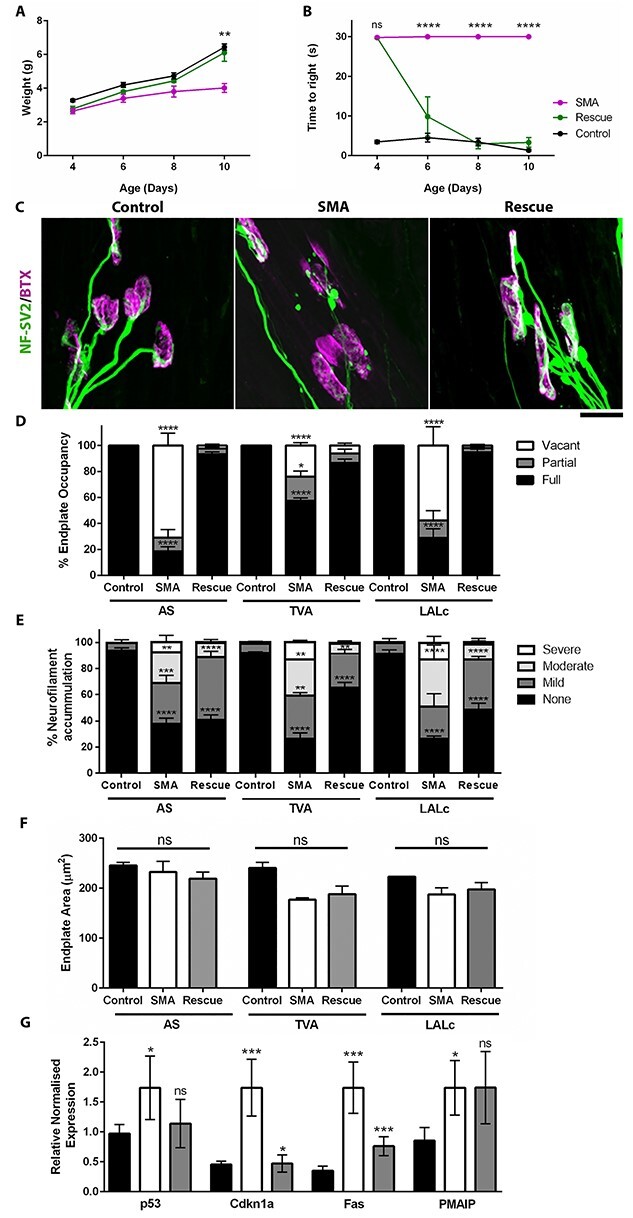
Restoration of Smn levels at P4 results in a significant rescue of NMJ pathology by P10. (**A**, **B**) Line chart (mean$\pm$SEM) showing body weight (A) and time taken to self right (B) in Rescue (Smn^Res/Res^;SMN2;SMNΔ7;Cre^+/−^), SMA (Smn^Res/Res^;SMN2;SMNΔ7;Cre^−/−^) and control mice at P4, 6, 8 and 10 days of age following treatment with Tamoxifen at P4. (**C**) Images showing neuromuscular junctions (NMJs) in the AS muscle labelled with antibodies against NF and SV2 (green) and BTX (magenta) in Rescue, Control and SMA mice at P10. Note presence of denervated endplates in SMA which was significantly reduced in Rescue mice. Scale bar = 20 μm. (**D**) Bar chart (mean ± SEM) showing percentage of fully occupied, partially occupied and vacant endplates in AS, TVA and Levator Auris Longus Caudal (LALc) muscles in Control, SMA and Rescue mice at P10. Stats show significant increase in percentage of fully occupied endplates in Rescue mice compared with SMA. (**E**) Bar chart (mean ± SEM) showing percentage of NMJs with no, mild, moderate or severe swelling in AS, TVA and LALc muscles in Control, SMA and Rescue mice at P10. Stats show comparison of percentage of NMJs with no swelling, comparing Rescue to SMA. (**F**) Bar chart (mean ± SEM) showing average endplate area in AS, TVA and LALc muscles in Control, SMA and Rescue mice at P10. (**G**) Bar chart (mean ± SEM) relative transcript levels of P53, Cdkn1a, Fas and Pmaip in the spinal cord of P10 Control, SMA and Rescue mice. Stats show SMA versus control and Rescue versus SMA. Note significant reduction in Cdkn1a and Fas in Rescue Mice compared with SMA compared with control. ^*^P < 0.05; ^*^^*^P < 0.01, ^*^^*^^*^P < 0.001, ^*^^*^^*^^*^P < 0.0001 by two-way ANOVA (A,B), one-way ANOVA with Tukey’s post hoc test (F) or Kruskal–Wallis with Dunn’s Post Hoc (D, E, G). *N* = 12/3/3 mice per Control/Rescue/SMA (A, B); *N* = 6/3/6 muscles per Control/SMA/Rescue (D–F) and *N* = 3 mice per genotype (G).

Previous work has shown that an increase in transcripts associated with the P53 signalling pathway correlates with the onset of NMJ pathology (14,29). We therefore quantified the levels of P53 and three transcripts known to be downstream of P53 (*Fas*, Pmaip and Cdkn1a) in spinal cord of P4 Smn^Res/Res^ mice. This revealed a significant increase in all four transcripts (Fig. 1F).

Collectively, this work reveals that at P4, a time point when induction of Smn levels can confer a significant extension in life span, there are already significant motor deficits, high levels of NMJ pathology in at least three vulnerable muscles and activation of the P53 signalling pathway in the spinal cord.

### Restoration of Smn levels at P4 ameliorates NMJ pathology and reverses activation of P53 signalling pathway

Our analysis at P4 revealed significant neuromuscular pathology at P4. We next investigated the extent to which this pathology is reversible, and the time frame in which this happens. Tamoxifen was administered by oral gavage at P4 (75 mg/kg) to induce full length expression of *Smn* in *Smn^Res/Res^;SMN2;SMN^Δ7;^Cre-ER^+/−^* mice (herein referred to as ‘Rescue’). *Smn^Res/Res^;SMN2;SMN^Δ7^* mice were used as non-induced SMA mice (herein referred to as ‘SMA’) and Smn^+/−^;SMN2;SMN^Δ7;^Cre-ER^+/−^ or Smn^+/+^;SMN2;SMN^Δ7;^Cre-ER^+/−^ were used as non-SMA controls (herein referred to as ‘Control’). Assessment of body weight revealed a trend towards an increase at P6 and P8, which was statistically significantly compared with ‘SMA’ mice, and indistinguishable from ‘Control’ mice by P10 (Fig. 2A). A significant improvement in motor performance was noted by P6, as evidenced by a decrease in the time taken to right from a prone position in ‘Rescue’ mice compared with SMA (Fig. 2B). Analysis of NMJ pathology revealed a dramatic reduction in NMJ loss compared with the same measurements taken at P4 and ‘SMA’ mice at P10 (Fig. 2C–F). Over 93, 87 and 95% of endplates were classified as fully occupied in AS, TVA and LALc, respectively, compared with 19, 57 and 29% in ‘SMA’ mice at P10. There was a significant but incomplete rescue in presynaptic swelling in the TVA and LALc muscles, but no significant improvement in the AS muscle. There was no significant change in endplate size in either AS, TVA or LALc in ‘Rescue’ mice compared with control or SMA (Fig. 2F).

Since q-RT-PCR analysis revealed a significant increase in P53 and downstream transcripts at P4, we investigated whether these changes were reversible. We observed a significant reduction in *Cdkn1a* and *Fas* in ‘Rescue’ mice compared with ‘SMA’, although Pmaip levels remained elevated (Fig. 2G). Collectively, this analysis demonstrated that many, but not all, aspects of neuromuscular pathology evident in a mouse model of SMA are reversible within a 6-day time frame.

### Motor unit size is preserved following restoration of Smn levels at P4 in *Smn*^*Res/Res*^ mice

**Figure 3:**
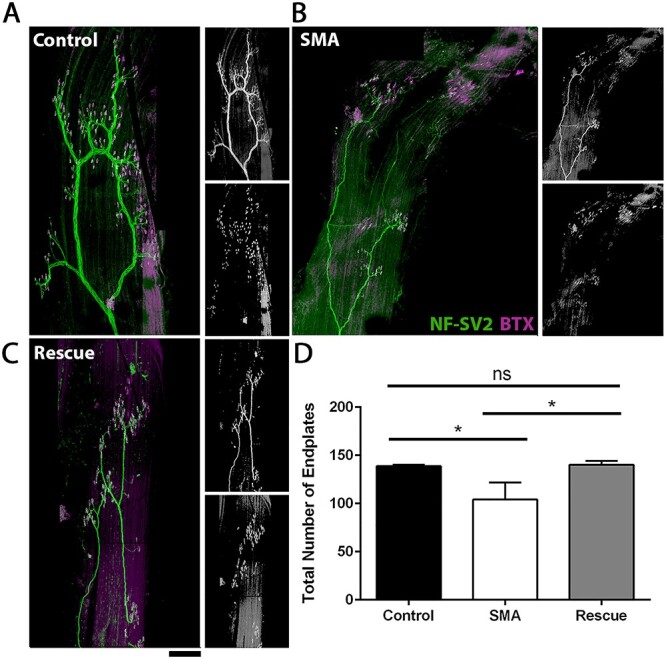
Recovery from denervation is due to reinnervation of denervated endplates. (**A**–**C**) Montages fluorescent micrographs showing C2 region of LALc muscle labelled with antibodies against NF and SV2 (green) and BTX (magenta) in Control (A), SMA (B) and Rescue (C) mice at P10. Scale Bar = 200 μm. (**D**) Bar chart (mean$\pm$SEM) showing significant decrease in total endplate number in SMA compared with both Control and SMA, but no difference in total endplate number in Rescue versus Control. ^*^P < 0.05; ns, non-significant by Student’s *t* test. *N* = 6/5/6 muscles per Control/SMA/Rescue.

Data presented in Figure 2 suggest that innervation can be restored following induction of full length Smn. However, it remained unclear whether the majority of endplates appear fully occupied because denervated muscle fibres have been reinnervated, or because denervated endplates have been lost. To address this question, we quantified total endplate numbers in the LALc muscle. This muscle was chosen as it has a well-characterized anatomy and is small and thin enough to whole mount, allowing analysis of the entire muscle without the need for sectioning. Previous work has revealed a consistent endplate band in LALc, at the medial side of the muscle, denoted C2 (17,30). Quantification of total endplate number in C2 revealed no significant difference between ‘Control’ and ‘Rescue’ mice, suggesting that the increase in endplate occupancy was a result of reinnervation rather than loss of denervated endplates (Fig. 3). Interestingly, there was a significant decrease in the number of endplates in the LALc muscle in ‘SMA’ mice compared with both control and rescue, suggesting that endplates which remain denervated are lost (Fig. 3). This finding reveals that assessing NMJ pathology by occupancy count alone, a practice which is widespread in the field of neuromuscular disease, can dramatically underestimate the levels of degeneration present.

Data thus far demonstrate the requirement for significant regeneration following Smn restoration. Such regeneration can be achieved via terminal sprouting and motor unit enlargement from remaining motor neurons, or via regeneration of degenerating axons. Although the former would correlate with an increase in the size of the motor unit, the later would result in a normal motor unit size. To distinguish between these possibilities, we have quantified axon number and extrapolated average motor unit size. Since axon number can vary along the length of a nerve, this analysis was performed in the AS muscle, a muscle in which the point at which two incoming bundles of axons cross over in a highly consistent manner. This allows us to quantify axon number at a consistent location in all muscles.

**Figure 4:**
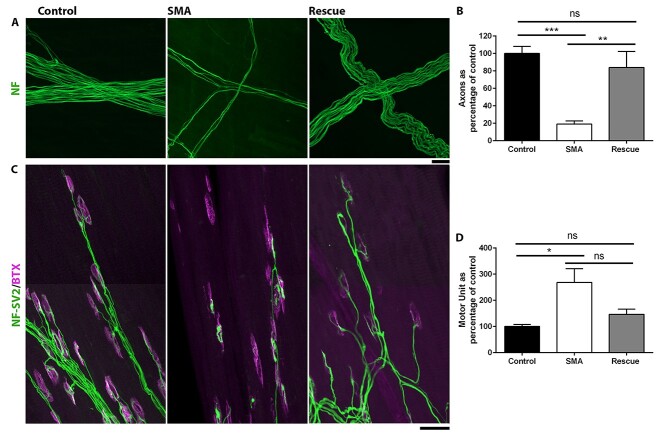
Smn restoration at P4 preserves axon number and motor unit size. (**A**, **C**) Images showing axon bundles (A) and neuromuscular junctions (NMJs) (C) labelled with antibodies against NF and SV2 (green) and BTX (magenta) in control, SMA and Rescue mice at P10. Scale bar = 20 μm (A) and 50 μm (C). (**B**, **D**) Bar chart (mean$\pm$SEM) showing number of axons (B) or average motor unit size (D) in AS muscle in Control, Rescue and SMA mice at P10. Note decrease in axon number and increase in motor unit size in SMA which was not seen in Rescue mice ^*^P < 0.05; ^*^^*^P < 0.01, ^*^^*^^*^P < 0.001, ns non-significant by Student’s *t* test (B) or Mann–Whitney U test (C). *N* = 5/6/5 muscles per Control/SMA/Rescue.

Quantification of axon number revealed that although there was a significant loss of axons in the AS muscle in ‘SMA’ mice, the number of axons in the ‘Rescue’ mice was not different to control levels (Fig. 4A and B). By dividing the number of innervated NMJs by the number of axons, we extrapolated the average motor unit size. This revealed that although there was a significant increase in the average motor unit size in ‘SMA’ mice, in Rescue mice, average motor unit size was not significantly different to ‘Control’ levels (Fig. 4D).

Collectively, these data show that restoration of Smn levels can result in remarkable protection of the motor unit, even after significant loss of NMJs and pathology within the motor unit. In addition, if Smn levels are restored before significant motor neuron loss, re-innervation can re-establish a normal motor unit size, correlating with a dramatic extension in lifespan.

### Delayed treatment with an ASO targeting SMN2 leads to widespread loss of axons and endplates

To investigate motor unit recovery using a more clinically relevant approach, we compared motor unit pathology following restoration of Smn levels using ASO targeting Element 1 of SMN2 [ASO^E1v1.11^; (31)] in the delta7 SMA mouse model. Previous work has routinely shown a dramatic extension in lifespan following SMN induction, using both genetic and viral approaches, before P2, with median lifespan of over 200 days (10–12). In contrast, mice in which SMN levels are increased at P6 or later have a median lifespan of between 30 and 35 days (10–12). This indicates that important changes occur between P2 and P6 which limit the effective therapeutic time window. To allow equivalent time for regeneration, this analysis was performed 11 days after ASO treatment i.e. P12 in P1-treated mice and P17 in P6-treated mice. As in other mouse models of SMA, intermuscular variability to pathology is a prominent feature in this mouse model, with different muscles following distinct time courses of degeneration. Therefore, to get a thorough overview of motor neuron recovery, we analysed three differentially vulnerable muscles: levator auris longus rostral (LALr, resistant), adductor auris longus (AAL, moderately affected) and AS (severely affected (Supplementary Material, Fig. S1). All muscles benefit from being small, thin and flat allowing a comprehensive analysis of innervation patterns.

**Figure 5:**
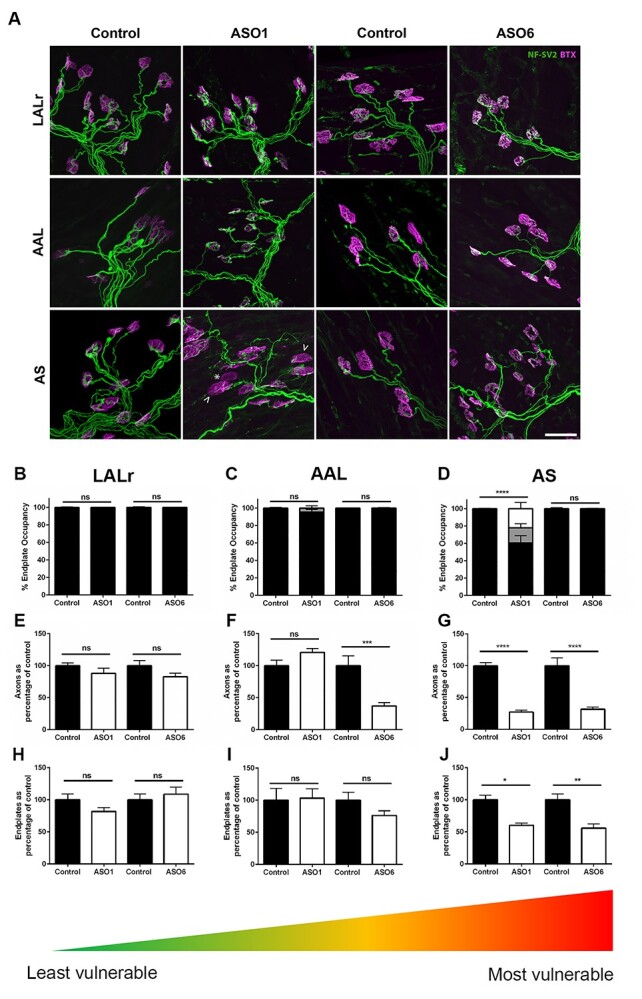
Delayed treatment with a Smn-inducing ASO leads to more widespread loss of axons and endplates. (**A**) Confocal images showing NMJs labelled with antibodies against NF and SV2 (green) and BTX (magenta) in SmnΔ7 mice treated with ASO at P1 (ASO1) or P6 (ASO6) with their respective controls. Muscles used are the LALr (least vulnerable), AAL (moderately vulnerable) and AS (most vulnerable). Vacant and partially occupied endplates in the AS after early treatment (ASO1) are marked by an asterisk and arrow heads, respectively. Scale bar = 20 μm. (**B**–**D**) Bar charts (mean ± SEM) showing the percentage of fully occupied (black), partially occupied (grey) or vacant (white) endplates in P12 Smn^−/−^;SMN2;SMNΔ7 mice treated with ASO at P1 (ASO1) or P6 (ASO6) compared with similarly treated controls. Muscles used are the LALr (least vulnerable), AAL (moderately vulnerable) and AS (most vulnerable). (**E–J**) Bar charts (mean ± SEM) showing the number of axons (E–G) and innervated NMJs (H–J) in the three muscle groups in P12/P17 Smn^−/−^;SMN2;SMNΔ7 mice following early (ASO1) or delayed (ASO6) treatment compared with controls. Note the significant loss of axons in the moderately vulnerable AAL muscle following delayed P6 treatment which was not seen in the early P1-treated cohort. Significant loss of both axons and endplates was seen in the severely vulnerable AS muscle following both P1 and P6 treatment. ^*^P < 0.05; ^*^^*^P < 0.01, ^*^^*^^*^P < 0.001, ^*^^*^^*^^*^P < 0.0001, ns non-significant by Mann–Whitney U test. *N* = 3–10 muscles per group.

To determine whether levels of innervation correlated with day of ASO administration, we first quantified the percentage of fully occupied endplates 11 days after P1 or P6 ASO administration, respectively (Fig. 5B–D). As expected, all endplates in the LALr were fully occupied. Interestingly, most endplates in AAL and AS muscles were also fully innervated after both P1 and P6 treatment, with the exception of a significant number of partially occupied and denervated endplates remaining in the AS following P1 treatment (Fig. 5D). Intriguingly, analysis of degree of NF accumulation revealed a similar trend (Supplementary Material, Fig. S2).

Since quantification of the percentage of fully occupied endplates alone can mask axonal and endplate loss (c.f. Fig. 4D), we next quantified axon and endplate numbers following P1 and P6 ASO treatment. Once again, there was no significant difference in the number of axons or endplates in the LALr muscle, verifying that pathology has not been masked by endplate fading or sprouting, and this muscle is truly resistant (Fig. 5E and **H**). In the moderately affected AAL muscle, although P1 treatment preserved axon and endplate number, following P6 treatment, there was a significant decrease in axon number (Fig. 5F and I). In the severely affected AS muscle, there was a significant loss of both axons and endplates following both P1 and P6 treatment. This work highlights the importance of considering multiple muscles and demonstrates each muscle follows its own degenerative timeline. It suggests that although a P1 intervention is adequate to rescue moderately affected muscles, for severely affected muscles, significant damage is present at P1 which was not reversed by ASO treatment.

### Delayed treatment with a Smn-inducing ASO results in a significant increase in motor unit size in a range of muscles

**Figure 6:**
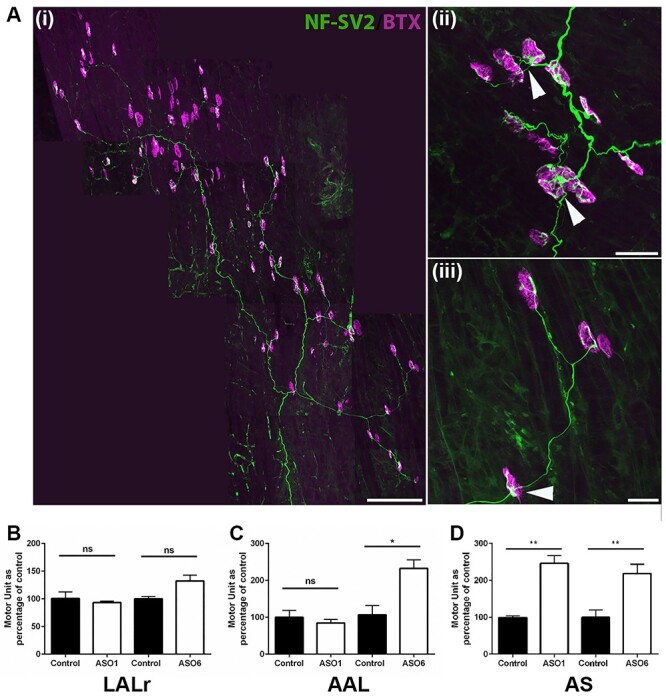
Axonal sprouting leads to increased motor unit size in both vulnerable and moderately resistant muscles following ASO treatment. (**A**) Confocal images showing NMJs labelled with antibodies against NF and SV2 (green) and BTX (magenta) in the AS muscle of a P12 Smn^−/−^;SMN2;SMNΔ7 mouse treated with ASO at P1. Note presence of large motor units (Ai) and evidence of significant presynaptic remodelling and terminal sprouting (Aii and (iii) respectively). Scale bar = 150 μm (Ai), 20 μm (Aii and Aiii). (**B**, **D**) Bar charts (mean ± SEM) showing average motor unit size in LALr, AAL and AS muscles of mice treated with ASO at P1 (ASO1) or P6 (ASO6) compared with similarly treated controls. Note that the significant loss of axons in the moderately vulnerable AAL muscle is masked by compensatory sprouting leading to enlarged motor units (B). ^*^P < 0.05; ^*^^*^P < 0.01, ^*^^*^^*^P < 0.001, ^*^^*^^*^^*^P < 0.0001, ns non-significant by Mann–Whitney U test. *N* = 3–10 muscles per group.

Since we observed a decrease in axon number in the absence of denervated endplates in the moderately affected AAL, we hypothesized that motor unit sprouting was compensating for some denervation following ASO administration. Qualitative analysis of AS muscles 12 days after ASO treatment revealed widespread evidence of sprouting (Fig. 6A). Indeed, in some muscles analysed only one or two motor units remained, allowing the entire motor unit to be traced. Examples of large motor units were noted, with size frequently around double the average motor unit size of controls (Fig. 6Ai). There was widespread evidence of terminal sprouting, where new axonal growth extends from the presynaptic terminal to reinnervated endplates which presumably had become denervated (Fig. 6Aii–iii).

To investigate the effect of this compensatory sprouting on the motor unit, we extrapolated average motor unit size by dividing the total number of innervated endplates by the number of axons entering the muscle. As expected, motor unit size was stable in the LALr muscle, (Fig. 6B). In the AAL muscle, although P1 ASO treatment preserved motor unit size, delayed P6 treatment led to a 2.2-fold increase in average motor unit size (Fig. 6C). In the vulnerable AS muscle, motor unit size was significantly increased following both P1 and P6 treatment (Fig. 6D). Collectively, these data demonstrate that following delayed administration of the ASO, the neuromuscular system is capable of dramatic repair, with considerable compensatory sprouting resulting in the reinnervation of denervated endplates in a relatively short time frame. However, regeneration results in a dramatic increase in motor unit size, which correlates with a poor phenotypic outcome.

## Discussion

The data presented here highlight the enormous regenerative capacity of the motor neuron following Smn restoration. Its impressive ability to recover from high levels of denervation in a comparatively short time frame is very encouraging, especially for patients treated after symptom onset. Preserving axon and endplate number and retaining a normal motor unit size all correlated with a dramatic extension in lifespan. Importantly, due to the previously unappreciated rapid loss of denervated endplates, the widely used technique of quantifying the percentage of fully occupied endplates significantly underestimates the levels of presynaptic degeneration. Our results also revealed that following ASO treatment and in untreated SMA mice, there was loss of axons and endplates and an increase in motor unit size, which was more prevalent following a delay in ASO administration. Investigating factors which can support large motor units throughout the lifespan of the individual may therefore represent an important therapeutic strategy for patients treated with a Smn-dependant therapeutic.

### Extent of recovery at the NMJ

Results here highlight the remarkable regenerative capacity of the NMJ, but also reveal that some NMJ defects persist following Smn restoration. Understanding the impact of these anomalies upon NMJ function and motor neuron health will be critical. Following genetic restoration of Smn levels, presynaptic swelling at the neuromuscular junction remains, the impact of which is unknown. Presynaptic swelling has been reported in muscles which are apparently resistant to pathology, suggesting it has minimal impact upon physiology. However, since mouse models of SMA have a significantly curtailed lifespan, the impact of these defects over a prolonged period has not been evaluated. It will therefore be important to determine whether similar defects exist in a human population, whether they resolve in the long term and what impact they have upon motor neuron physiology over a prolonged period of time.

Following ASO administration, there was a loss of axons and endplates, which occurred in some muscles even when ASO was given at P1. The reason why rescue was less complete following ASO treatment, compared with the genetic rescue is unknown. It is possible that this is because of the higher Smn levels achieved in the genetic rescue; however, unfortunately, this was not something that we were able to assess. Regardless, this work highlights that following Smn restoration, especially after ASO treatment, recovery is incomplete. Understanding the basis for these defects, and the reasons why they are not corrected, could be the basis for combinatorial therapeutics to aid NMJ recovery and aid the long-term motor neuron health following Smn-dependant therapeutic administration in SMA.

### Denervation induced loss of motor endplates

Here we show that, following presynaptic pathology and a loss of presynaptic inputs, motor endplates are lost. Endplates initially appear visibly fainter following labelling with alpha-bungarotoxin (BTX), suggesting they are progressively disassembled following loss of presynaptic input. Previous work has often reported that although there is a significant increase in denervated endplates in the early symptomatic phases in mouse models of SMA, the severity of denervation does not increase as pathology progresses (16,17). The data presented here suggests that this could be due to the loss of endplates as they become denervated, rather than the cessation of denervation. The quantification of the percentage of fully occupied endplates is standard practise for assessing the degree of presynaptic degeneration occurring in SMA and other peripheral neuropathies. However, these current findings highlight how this can grossly underestimate, and even mask, levels of presynaptic degeneration. These data therefore invite careful consideration as to the methods used to quantify presynaptic loss so that it is accurately represented.

To our knowledge, the fading and loss of endplates in models of motor neuron disease have not been previously characterized. Endplate stability has been studied in experimentally denervated muscles, with significant remodelling of the AChRs, fading and loss of chronically denervated receptor clusters reported following nerve injury (32). However, Kang *et al*. also showed that persistent clusters of AChRs remain over 20 days after denervation. The loss and fading of AChR clusters in this study appear to occur at a much more rapid rate. This could be due to the comparatively immature status of endplates in mouse models of SMA (17,33), as the rate of AChR turnover is known to be dramatically increased during post-natal development (34,35). Alternatively, the rapid loss of endplates could indicate an intrinsic defect in AChR maintenance caused by a deficiency in Smn. Tight control of actin dynamics is thought to have an important role in stability and turnover of AChRs, particularly during postnatal development (35–38). Since defects in the actin cytoskeleton have been widely reported in mouse models of SMA [e.g. (39–43)], it is tempting to speculate that this could contribute to the rapid loss of AChR clusters following denervation. Distinguishing these possibilities will be vital in determining the importance and translational relevance of AChR receptor loss following denervation.

### Implications of motor unit enlargement in SMA

Due to sprouting from remaining axons, the average motor unit size can increase following Smn restoration. This was particularly evident following a delay in treatment. Motor unit enlargement has been previously described in SMA patients and in asymptomatic mouse models with reduced Smn levels. Although in the short term, an increase in motor unit size can compensate for denervation and slow disease progression, larger motor units are thought to encounter heightened levels of oxidative stress, because of their larger metabolic demand. Indeed, many individuals who experienced motor neuron loss due to acute polio infection in childhood have gone on to experience muscle weakness, fatigue and atrophy in adulthood, even in muscles which were apparently not affected during the acute infection (44,45). This has been termed post-polio syndrome and is thought to be caused by the premature aging and loss of large motor units which underwent compensatory enlargement during childhood (45). This may have particular relevance to SMA patients treated with a Smn-dependent therapeutic, and it will be important to monitor motor ability throughout their lifespan and to consider the likely outcome for enlarged motor units and how to best support them throughout the lifespan of the individual.

## Material and Methods

### Mouse breeding and maintenance

*Smn^+/Res^;SMN2;SMNΔ7;Cre^ERT^* mice (strain 008783, mixed C57BL/6;FVB/N;129 genetic background) were purchased from Charles River and maintained as *Smn^+/Res^;SMN2;SMNΔ7;Cre-ERT^+/−^* × *Smn^+/Res^;SMN2;SMNΔ7* breeding pairs at the animal facilities at the University of Edinburgh. *Smn^+/−^;SMN2^+/+^;SMNΔ7^+/+^* (Strain 005025, Strain FVB/N) were maintained as breeding pairs at the University of Missouri. All genotyping was performed using standard PCR protocols on DNA extracted from an ear punch (for breeding pairs), tail tip (taken post-mortem for experimental mice) or tail sample (taken before tamoxifen treatment for tamoxifen treated mice). Mice over P10 were sacrificed by overdose of inhalation anaesthetic or rising CO_2_ and death was confirmed by exsanguination from the carotid artery. Mice under P10 were decapitated under terminal anaesthesia and death was confirmed by exsanguination from the carotid artery. All experiments were performed in accordance with the regulations set out by the UK Home Office (for animal experiments performed in the UK) or approved by the IACUC at the University of Missouri.

### Administration of tamoxifen

Tamoxifen (Sigma-Aldrich) was dissolved in corn oil to a concentration of 20 mg/ml. Tamoxifen solution was administered to litters of mice at P4 using oral gavage at a dose of 75 mg/kg. For Figures 2–4, all mice in a litter received tamoxifen. Cre-negative mice (i.e. Smn^Res/Res^;SMN2;SMNΔ7) were used as SMA model mice, as no recombination will take place, and mice carrying one copy of the Cre transgene (i.e. Smn^Res/Res^;SMN2; SMNΔ7;Cre-ERT^+/−^) were used as ‘Rescue’ mice.

### Antisense oligonucleotide treatment

P1 SMNΔ7 mice were injected with 4 μL of a 20 nM solution of E1^v1.11^, a Phosphordiamidate morpholino oligomer (PMO) ASO (31). The following primer sets were used: for the mouse *Smn* gene, mSmn-Wildtype (WT) forward (5′-tctgtgttcgtgcgtggtgacttt-3′) and mSmn-WT reverse (5′-cccaccacctaagaaagcctcaat-3′) and for the *Smn* knockout, SMN1-KO forward (5′-ccaacttaatcgccttgcagcaca-3′) and SMN1-KO reverse (5′-aagcgagtggcaacatggaaatcg-3′). intracerebroventricular (ICV) injections were performed on P1 and the injection site was $\sim$0.25 mm lateral to the sagittal suture and 0.50–0.75 mm rostral to the neonatal coronary suture. The needles were inserted perpendicular to the skull surface using a fiber-optic light (Boyce Scientific Inc.) to aid in illuminating pertinent anatomical structures. Needles were removed after 10 s of discontinuation of plunger movement to prevent backflow. Treated animals were placed in a warmed container for recovery (5–10 min) until movement was restored.

### Motor testing

The ‘time to right’ test was carried out on mice younger than P10. Mice were placed on their back and the time it took for them to right themselves (as defined by the placement of all 4 paws on the testing surface) was recorded, with a maximum of 30 s and an average of three attempts.

### NMJ labelling

Muscles were immediately dissected from recently sacrificed mice and fixed in 4% paraformaldehyde (Electron Microscopy Science) in PBS for 15 min. Post-synaptic AChRs were labelled with α-BTX (1:250; tetramethylrhodimine conjugate, Life Technologies) for 2 h. Muscles were permeabilized in 2% Triton X-100 in phosphate buffered saline (PBS) for 30 min, then blocked in 4% bovine serum albumin/1% Triton X-100 in PBS for 30 min before incubation overnight in primary antibodies [NF, 1:50 (2H3)—Developmental Studies Hybridoma Bank; synaptic vesicle protein 2, 1:100 (SV2)—Developmental Studies Hybridoma Bank] and visualized with secondary antibodies (AlexaFluor-488 rabbit anti-mouse at 1:250, Jackson). Muscles were then whole-mounted in Mowiol (Sigma).

The percentage of fully occupied endplates was determined by classifying each endplate in a given field of view either fully occupied [presynaptic terminal (SV2 and NF) completely overlies endplate (BTX)], partially occupied [presynaptic terminal only partially covers endplate (BTX)] or vacant (no presynaptic label overlies endplate). For presynaptic swelling, all NMJs were classified as having no swelling, mild (evidence of some axonal swelling and/or presynaptic swelling that does not obscure the end plate), moderate (evidence of clear axonal swelling and significant presynaptic swelling that is beginning to obscure the end plate) or severe (significant and obvious swelling along the length of the axon and severe swelling around the presynaptic terminal, which obscures the endplate). Endplate size was quantified using ImageJ software. At least three fields of view were analysed per muscle totalling > 50 endplates per muscle. Endplate number was assessed by counting the number of AChR clusters within an anatomically consistent area of the muscle (LALc, C2; AS and AAL, entire medial endplate band). NMJ numbers were assessed by counting the number of fully or partially innervated endplates in an anatomically distinct area of the muscle. Only muscles which were present in their entirety with no signs of damage or disruption to innervation patterns were used for these measurements. Axon number was counted in a consistent region of each muscle (the point at which axons cross in the AS and 400 μm from the main endplate band in the AAL). Average motor unit size was calculated by dividing the total number of innervated NMJs in a given muscle by the number of axons in the same muscle. All quantification was performed with researcher blind to genotype of material.

### q-RT-PCR

RNA was extracted using a micro RNeasy kit (Qiagen) and 1 μg of RNA was used to perform reverse transcriptase using the RT^2^ First Strand kit (Qiagen). SYBR green-based Q-RT-PCR was performed using preoptimized primers purchased from Qiagen. Amplification was performed using KAPA SYBR fast universal PCR mastermix as per manufacturer instructions on a BioRad CFX connect real-time PCR detection system. Relative gene expression was calculated using the 2^−ΔΔcT^ formula (46). Relative levels are expressed normalized to GusB (F: GGCTGGTGACCTACTGGATTT; R: TTGGCACTGGGAACCTGAAGT) and YWHAZ (F: TTGATCCCCAATGCTTCGC; R: CAGCAACCTCGGCCAAGTAA).

## Supplementary Material

Supp_figure_1_ddac097

Supp_figure_2_ddac097
