# Supplementary figures and images for: Motor unit recovery following Smn restoration in mouse models of spinal muscular atrophy
Source: Hum Mol Genet. 2022 May 12;31(18):3107–19. doi: 10.1093/hmg/ddac097 (PMC9476628; doi:10.1093/hmg/ddac097)

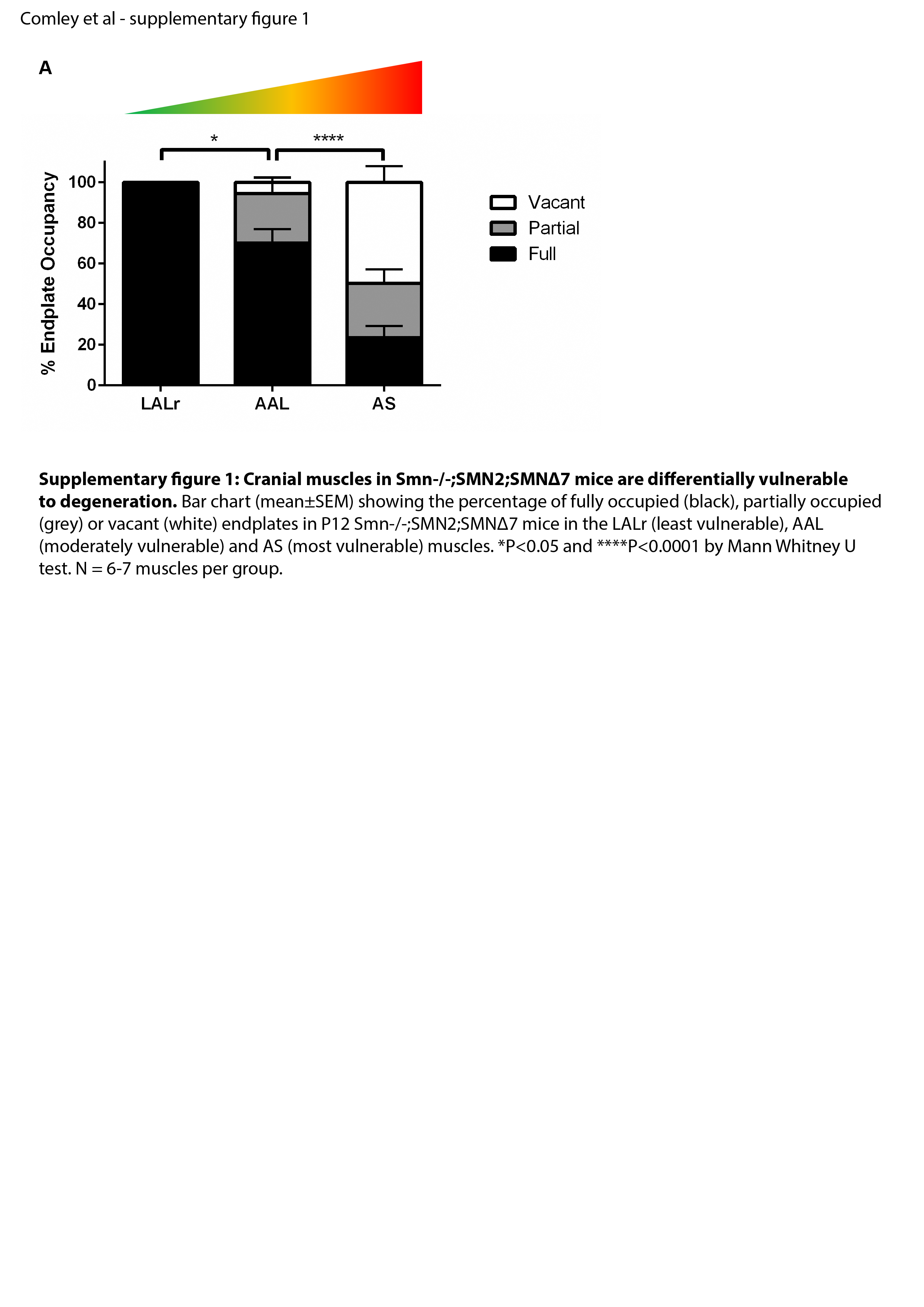

Supplement: Supp_figure_1_ddac097 [file supp_figure_1_ddac097.zip › Supp_figure_1_ddac097.tif]

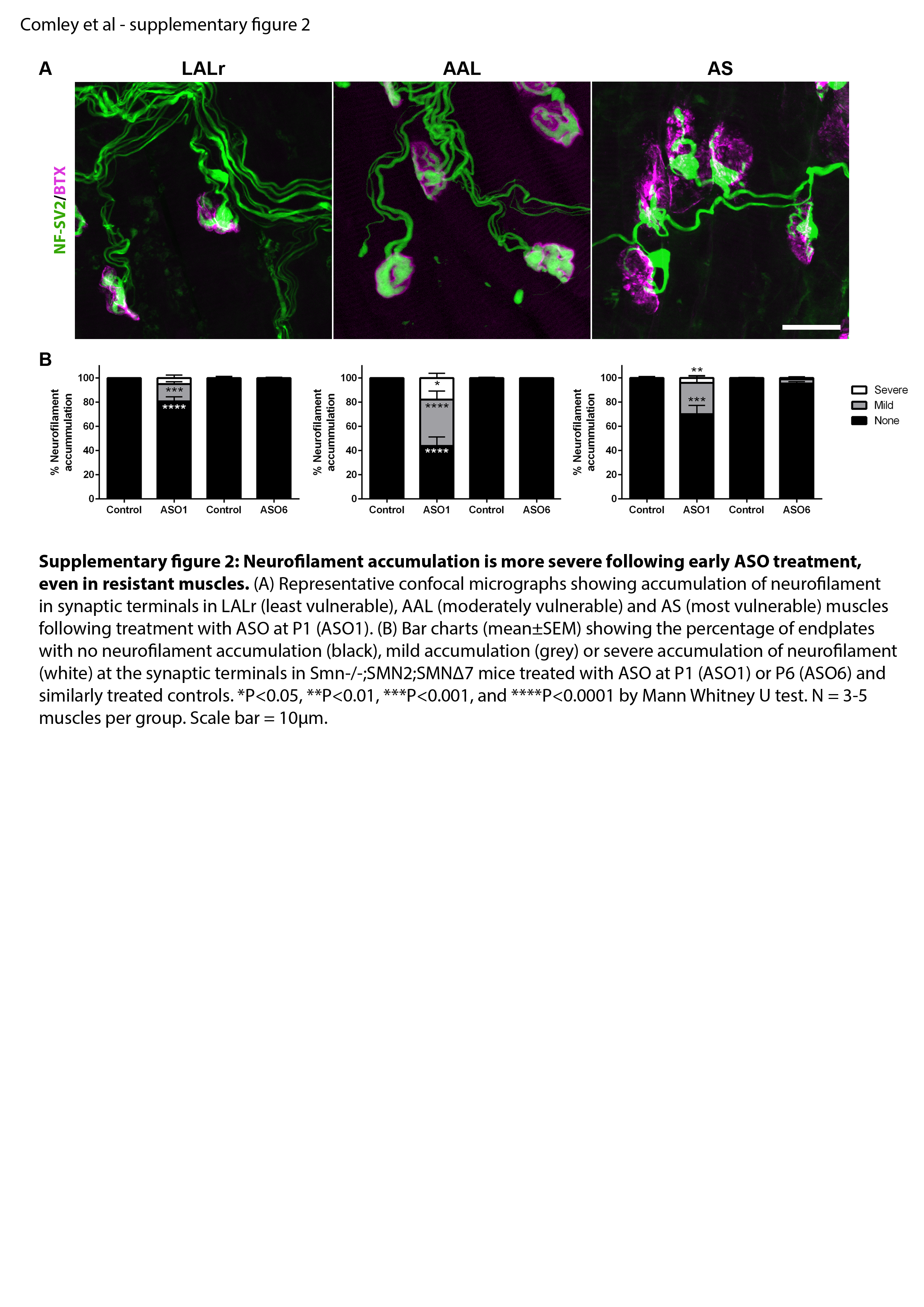

Supplement: Supp_figure_2_ddac097 [file supp_figure_2_ddac097.zip › Supp_figure_2_ddac097.tif]
